# Effects of Palladium Precursors on the Activity of Palladium Nanocatalysts for the Oxidation of Volatile Organic Components

**DOI:** 10.3390/nano13071189

**Published:** 2023-03-27

**Authors:** Qingtao Li, Qi Cai, Xiaoyun Li, Enshan Han, Yanmin Sun, Yanfei Lu, Zhe Cai, Haibin Yu

**Affiliations:** 1 School of Chemical Engineering and Technology, Hebei University of Technology, Tianjin 300401, China; 13406393690@163.com; 2 CNOOC Tianjin Chemical Research and Design Institute Co., Ltd., Tianjin 300131, China; caiqi168365@126.com (Q.C.); lixy49@cnooc.com.cn (X.L.); sunym8@cnooc.com.cn (Y.S.); luyanfei19870825@163.com (Y.L.); caizhe1991@126.com (Z.C.);

**Keywords:** volatile organic components, palladium, catalytic oxidation, precursors

## Abstract

To screen a suitable precursor, the effects of palladium salts on performance of Pd nanocatalysts for the oxidation of volatile organic components (VOCs) were investigated. A series of catalysts was prepared by impregnating Pd(NO_3_)_2_, PdCl_2_ and Pd(NH_3_)_4_Cl_2_ on alumina-coated cordierites. These catalysts were characterized by XRF, ICP-OES, XRD, N_2_ adsorption-desorption, TEM, EDS, Raman spectroscopy, pulse-CO chemisorption, H_2_-TPR, NH_3_-TPD, and XPS. Pulse-CO chemisorption and TEM showed that Pd species formed by Pd(NO_3_)_2_ have the highest metal dispersion (17.7%), while the other two were aggregating. For the same Pd loading, the higher the metal dispersion, the more the number of PdO species, so the number of PdO particles in the catalyst prepared from Pd (NO_3_) _2_ is the largest. The catalytic oxidation activities of these catalysts were evaluated by ethane and propane. Based on a 99% conversion in the oxidation of ethane and propane at 598 K and 583 K, respectively, the catalyst prepared from Pd(NO_3_)_2_ was considered to be the best performing catalyst. The chloride species in precursors can promote the aggregation of Pd species and poison the catalysts. The results show that Pd(NO_3_)_2_ is more suitable as the precursor of VOC oxidation catalyst than PdCl_2_ and Pd(NH_3_)_4_Cl_2_.

## 1. Introduction

Emission of volatile organic compounds (VOCs) is one of the major contributors to air pollution. VOCs refer to low molecular weight carbon-based molecules (up to C20). At normal temperature (273 K) and pressure (10^5^ Pa), the saturated vapor pressure of VOCs is higher than 70 Pa and the boiling point is lower than 533 K [1,2]. VOCs can be divided into alkanes, olefins, aromatic hydrocarbons, alkynes, halogenated hydrocarbons, etc. With the rapid development of industry, the emission of VOCs is increasing, which can bring negative health effects to humans and environment. Anthropogenic non-methane VOCs emissions in China have reportedly increased from 9.76 Tg in 1990 to 28.5 Tg in 2017 [3]. Therefore, numerous research initiatives have attempted to develop efficient technologies to reduce the emission of VOCs in the environment.

Among the various technologies for VOCs removal, catalytic oxidation is one of the most effective, economically feasible and widely used techniques in the world. Commonly, catalytic oxidation catalysts can be classified into two groups: supported noble metals catalysts and transition metal oxides catalysts [4,5,6]. Supported noble metals catalysts are widely used for catalytic oxidation of VOCs due to their advantages of low ignition temperature of 498 K and high catalytic activity [7,8]. It is reported that Pd catalysts possess high activity for the catalytic oxidation of light alkanes [9]. After introducing 24% TiO_2_ into Pd/Al_2_O_3_, Lin et al. [10] found that the temperature with methane conversion of 10% would decreased by 30 K. At about 383 K, the 100 ppm o-xylene was completely oxidized to CO_2_ and H_2_O over 1% Pd/Al_2_O_3_ at a gaseous hourly space velocity of 10,000 h^−1^ [11]. After hydrophobic modification of γ-Al_2_O_3_ carrier with triethoxyoctylsilane, Cui et al. [12] found that with the increase of surface Pd^0^ site concentration from 81.2% to 86.7%, the temperature with methane conversion of 90% decreased from 648 K to 623 K. Based on a simple one-pot, two-step method using Pd(NH_3_)_4_(NO_3_)_2_ as a starting material, Dong et al. [13] designed and prepared a novel pure silica zeolite silicalite-1 enveloping Pd-CeO_2_ nanowires (Pd-CeO_2_NW@S-1) catalyst to achieve an improved activity by reducing the reaction temperature for 90% VOC conversion to 569 K. In addition, the activity of Pd-CeO2NW@S-1 catalyst will hardly decrease after being reused for five times. By introducing 20% doping of Pt in PdCl_2_ raw material, Yang et al. [14] found that the Pd-Pt/γ-Al_2_O_3_ catalyst almost maintained the original catalytic combustion activity after sulfidation, while the catalytic combustion activity of the Pd/γ-Al_2_O_3_ catalyst decreased by 20%. Xiong et al. [15] prepared Pd-Co_2_NiO_4_ catalyst by embedding the PdO_x_ species into the Co_2_NiO_4_ lattice and loading it on the outer surface of Co_2_NiO_4_. At 610 K, the conversion of methane over the catalyst was as high as 99.2%. Chen et al. [16] found that the catalysts prepared from different Zr precursors have different phase structures. The use of ZrOCO_3_ or ZrO(NO_3_)_2_ as a Zr precursor gave Ce_0.45_Zr_0.45_La_0.1_O_1.95_ mixed oxides with a cubic phase, while an extra t″ phase was observed when Zr(NO_3_)_4_ was used as the Zr precursor. Among the three precursors, the Pd/Ce_0.45_Zr_0.45_La_0.1_O_1.95_ catalyst prepared using ZrOCO_3_ as Zr precursor exhibited the best textural, structural, and redox properties as well as the highest catalytic activity for the conversion of C_3_H_8_, CO, and NO. It was found that different Zr sources had a significant effect on the catalytic oxidation performance of the catalysts. However, a similar study on the effect of different Pd sources on the performance of VOC oxidation catalysts has not been reported.

In this paper, a series of Pd nanocatalysts is prepared by impregnating different palladium salts (Pd(NO_3_)_2_, PdCl_2_ and Pd(NH_3_)_4_Cl_2_) on alumina-coated cordierite with low thermal expansion high temperature resistance and low cost. The physical and chemical properties of these catalysts are characterized by X-ray fluorescence (XRF), inductively coupled plasma optical emission spectrometry (ICP-OES), X-ray diffraction (XRD), N_2_ adsorption-desorption, H_2_ temperature-programmed reduction (H_2_-TPR), the temperature-programmed desorption of NH_3_ (NH_3_-TPD), transmission electron microscopy (TEM), X-Ray photoelectron spectroscopy (XPS), and other methods. Ethane and propane with stable C-H bond are used as the model reactant and the catalytic oxidation activities of the catalysts are evaluated in a fixed bed reactor. The purpose of this paper is to explore the effect of Pd precursors on the activity of Pd nanocatalysts for the oxidation of VOCs.

## 2. Experiment

### 2.1. Materials

Palladium nitrate dihydrate (Pd(NO_3_)_2_·2H2O), palladium chloride (PdCl_2_) and tetraammine dichloropalladium monohydrate (Pd(NH_3_)_4_Cl_2_) are purchased from Aladdin Reagent Co., Ltd. (Shanghai, China). Nitric acid (HNO_3_, 68% *w*/*w*) are purchased from Damao Chemical Reagent Factory, Tianjin, China. All reagents were of analytical grade. Gamma-alumina and pseudo-boehmite were purchased from Shanxi Juhua New material Technology Co., Ltd., Shanxi, China. Cordierite honeycomb ceramics (31 mm × 31 mm × 25 mm, 200 holes per square inch, density is 500 kg/m^3^) were purchased from Kexing special ceramics Co., Ltd., Pingxiang, Jiangxi, China.

### 2.2. Preparation of Catalysts

The Pd nanocatalysts were prepared by an impregnation method. Firstly, 20 g pseudo-boehmite was dispersed in 40 g deionized water and 40 g HNO_3_ (3% *w*/*w*, diluted by 68% *w*/*w* HNO_3_) was added while stirring. The mixture was stirred evenly for 1h to obtain 100 g aluminum glue. Then, 180 g γ-Al_2_O_3_ powder was dispersed in 720 g deionized water, stirred evenly, and then 100 g aluminum glue was added to prepare the slurry with solid content of 20% *w*/*w*. Cordierites were soaked in deionized water and were ultrasonically treated to remove the powder and particles in the pores. Then they were dried in an oven at 393 K for 12 h.

The pretreated cordierites were impregnated into the slurry of γ-Al_2_O_3_ for 15 min. After blowing with compressed air for 5 min, the cordierites were dried at 393 K for 2 h. Then, they were calcinated in air at 873 K for 4 h with a heating rate of 2.5 K/min. Secondly, Pd(NO_3_)_2_, PdCl_2_ and Pd(NH_3_)_4_Cl_2_ solutions were prepared with a Pd^2+^ concentration of 1.0% *w*/*w*. The samples were correspondingly prepared using the above impregnation solution. The samples were dried at 393 K for 2 h and calcinated at 873 K for 4 h with a heating rate of 2.5 K/min. In this way, an 8% *w*/*w* γ-Al_2_O_3_ loading and a 0.2% *w*/*w* Pd loading were obtained in the cordierite. The three catalysts were named Pd-1, Pd-2 and Pd-3. Blank cordierite, γ-Al_2_O_3_, and cordierite coated with γ-Al_2_O_3_ were retained as a control, which were correspondingly named COR, γ-Al_2_O_3_, and Al_2_O_3_/COR.

### 2.3. Evaluation of Catalytic Oxidation Activities

The catalytic oxidation activities of the catalysts for ethane were evaluated in a fixed-bed reactor. The bulk catalyst (31 mm × 31 mm × 25 mm) was tested in a square reactor. The feed gas consisted of 0.2 vol% ethane, 21 vol% O_2_ and 78.8 vol% N_2_. The gas hourly space velocity (GHSV) was set at 10,000 h^−1^. The evaluated temperature range was from 523 K to 823 K. Gas chromatography (Agilent-7890) equipped with flame ionization detector (FID) and thermal conductivity detector (TCD) was used to test the feed gas and tail gas of the evaluation device. The catalytic effect is judged by the conversion of ethane, which was estimated by the expression below:

(1) $$\varphi_{C}=\frac{C_{\mathrm{in}}-C_{\mathrm{out}}}{C_{\mathrm{in}}}\times 100\%$$

where $\varphi_{C}$ is the conversion of ethane, $C_{\mathrm{in}}$ is the concentration of ethane in the feed gas of the unit, and $C_{\mathrm{out}}$ is the concentration of ethane in the tail gas of the unit.

### 2.4. Characterization of Catalysts

The bulk catalysts were crushed and particles with the size of 2–4 mm were collected. These particles were analyzed by H_2_-TPR, NH_3_-TPD, pulse CO chemisorption and XPS. The particles of catalysts were ground and the powder with <40 µm diameters was collected. They were analyzed using XRD, ICP-OES, and Raman spectroscopy. The catalysts powder was pressed into flakes with ~30 mm diameter and ~3 mm thickness before they were characterized by XRF. The catalysts powder was dispersed in ethanol and then ultrasonically dispensed into a suspension. The suspension droplets were allowed to dry before being used in TEM analysis.

Chemical analysis of catalysts was performed by XRF using a ARL 9800 spectrometer (Thermo Fisher Scientific, Waltham, MA, USA). The Pd content of catalysts was determined by ICP-OES on a Poridgy 7 spectrometer (Teledyne Leeman Labs, Hudson, NH, USA). Working conditions of the instrument: radio frequency generator power 1.1 kW, cooling gas (Ar) flow rate 18 L/min, atomization gas (Ar) flow rate 34 L/min, auxiliary gas (Ar) flow rate 0.2 mL/min, injection time 30 s.

XRD patterns were recorded using a Cu Kα (1.54 Å) radiation on a D/MAX x-ray diffractometer (Rigaku, Tokyo, Japan) at a voltage of 40 kV and a current of 200 mA. The catalysts were analyzed over a diffraction-angle (2θ) range of 5–80°, with scanning rate of 10°/min.

The texture properties of the samples were determined by low-temperature adsorption of nitrogen at 77 K using a Micromeritics ASAP2420 sorptometer (Atlanta, GA, USA). Specific surface area (S_BET_) was evaluated according to the Brunauer–Emmett–Teller (BET) method. Pore volume (V_BJH_), pore size distribution and mean pore size (D_BJH_) were calculated using the Barrett–Joyner–Halenda (BJH) method.

TEM images, high-resolution transmission electron microscopy (HRTEM) images and energy-dispersive X-ray spectroscopy (EDS) mapping were recorded on the Talos F200s high resolution transmission electron microscope (Thermo Scientific, MA, USA) equipped with an energy-dispersive X-ray spectroscope, operating at 200 kV. The samples were ground and dispersed into ethanol, and then deposited on ultra-thin carbon film for HRTME images and EDS mapping.

Catalysts were characterized by inVia Reflex microRaman spectroscopy (Renishaw, London, UK). The excitation source employed was a laser operated at 7.5 mW with a wavelength of 532 nm.

H_2_-TPR, NH_3_-TPD, and pulse CO chemisorption were measured by AutoChem II 2920 HP chemical adsorption apparatus (Micromeritics, ATL, USA).

For H_2_-TPR, approximately 300 mg catalyst sample was pretreated in an Ar flow (30 mL/min) at 473 K for 1 h and cooled to 333 K. The temperature increased to 973 K at 10 K/min in a flow of 10 vol% H_2_/Ar (30 mL/min). The hydrogen consumption was detected by the TCD detector (ATL, USA).

For NH_3_-TPD, about 100 mg catalyst sample was pretreated in an Ar flow (20 mL/min) at 423 K for 40 min and cooled to 323 K. Then Ar gas was switched to a mixture of 10 vol% NH_3_/He (40 mL/min) for 20 min. Next, a mixture of 10 vol% NH_3_/He was switched to He gas for 30 min. NH_3_-TPD signals were collected from 323 K to 1173 K at a rate of 10 K/min.

For pulse CO chemisorption, about 300 mg catalyst sample was cooled to 313 K in a He flow (20 mL/min) and wait until the baseline became stable. Then known amounts of CO were pulsed into the sample under a flow of inert gas. The amount of CO adsorbed in each pulse was quantified by analyzing the outlet gas concentration using a TCD. The pulses were repeated until the sample was fully saturated with the probe molecule.

XPS was recorded with a Thermo Scientific Escalab 250Xi instrument with Al Kα anode (1486.6 eV). The binding energy (BE) was corrected using the C1s photopeak at 284.8 eV as an internal standard. Peak fitting was processed with Casa XPS software in order to determine the peak position, height, and width.

## 3. Results and Discussion

### 3.1. Analysis of Catalysts Composition

The elemental type and content of Pd-1, Pd-2, and Pd-3 were analyzed by XRF. The results obtained are shown in Table 1. Pd-1, Pd-2, and Pd-3 have almost the same Pd loading. In addition, Cl element was found in Pd-2 and Pd-3. To verify the content of Pd in the catalysts, 0.5 g of Pd-1, Pd-2, and Pd-3 was correspondingly added to 10 mL of aqua regia. After 2 h to remove the residue. The residue was washed with deionized water three times. The combined solutions were diluted to 100 mL before measurements. The results are summarized in Table 1. Combined with the errors of ICP-OES results and preparation loss, dissolution, and dilution, it is considered that the Pd content of catalyst Pd-1, Pd-2, and Pd-3 is close to the target value (0.2% *w*/*w*).

### 3.2. Structural Characterization

The crystal structure of blank cordierite (COR), cordierite coated with γ-Al_2_O_3_ (Al_2_O_3_/COR) and catalysts Pd-1, Pd-2, and Pd-3 were examined by XRD.

**Figure 1:**
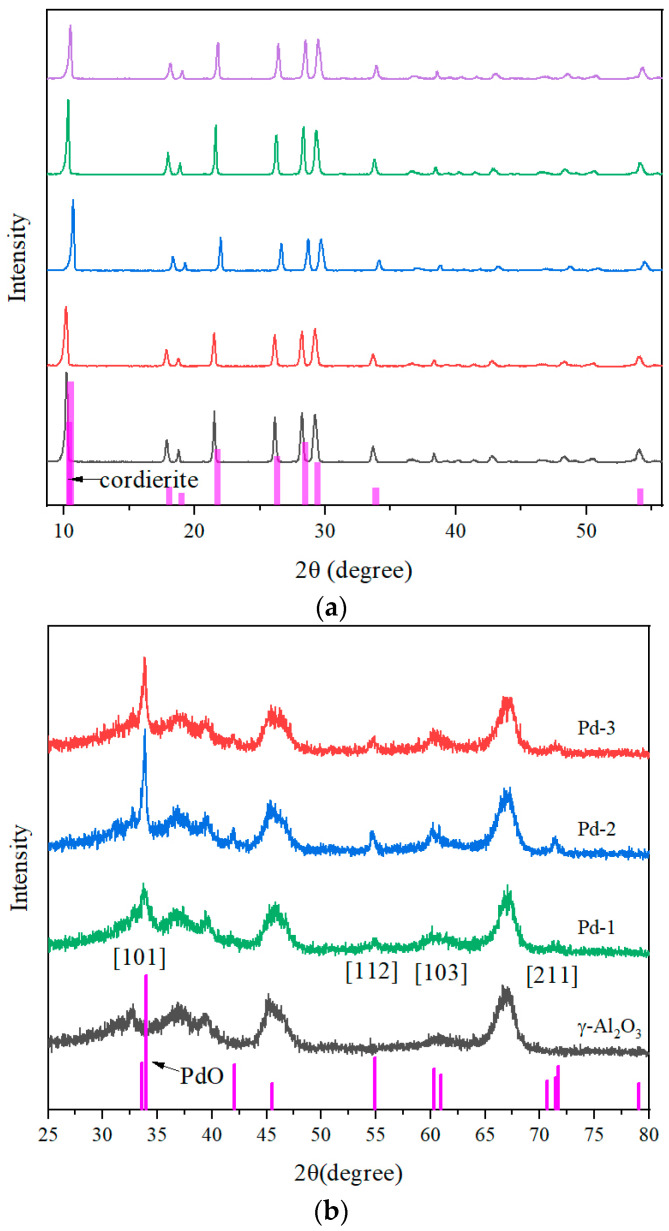
(**a**) X-ray diffraction patterns of COR, Al_2_O_3_/COR, Pd-1, Pd-2 and Pd-3. (**b**) X-ray diffraction patterns of γ-Al_2_O_3_, Pd-1, Pd-2 and Pd-3 de-cordierite samples.

In Figure 1a, Al_2_O_3_/COR and Pd-1, Pd-2, Pd-3 have similar diffraction peaks with the sample of COR, indicating that the coating of γ-Al_2_O_3_ and the loading of Pd would not alter the crystal structure of cordierite. A series of characteristic peaks of cordierite (ICSD/JCPDS#:85-1722) could be observed at 2 θ = 10.35°, 10.45°, 18.04°, 18.95°, 21.69°, 26.28°, 28.43°, 29.40°, 33.74°, and 54.09°, corresponding to [200], [110], [310], [002], [202], [312], [222], [421], [512], and [624] planes. In addition, no obvious diffraction peaks of Pd species were observed in the XRD patterns, which should be related to the low loading and the high dispersion of Pd species [17,18,19].

The coating on the catalysts was scraped off and characterized by XRD, and the results are shown in Figure 1b. With the background of γ-Al_2_O_3_, a series of characteristic peaks of PdO (ICSD/JCPDS#:75-0584) could be observed at 2θ = 33.9°, 54.9°, 60.4°and 71.7°, corresponding to [101], [112], [103], and [211] planes. Figure 1b shows that the intensity of the diffraction peak of Pd species is different among all three catalysts, with Pd-being the lowest, especially the peak at 33.9°. The reason is that the dispersion of Pd species on Pd-1 is the highest and the distribution is the most uniform.

**Figure 2:**
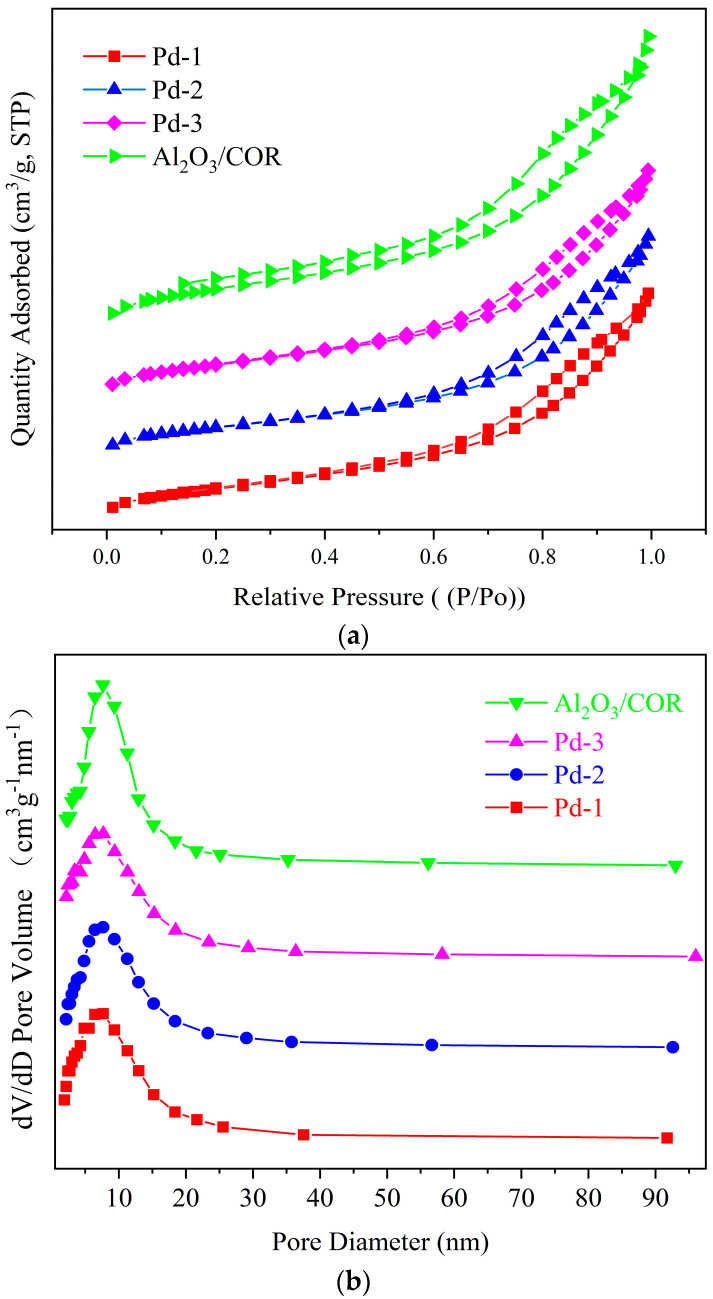
(**a**) N_2_ adsorption and desorption isotherms of Pd-1, Pd-2, Pd-3 and Al_2_O_3_/COR at 77 K. (**b**) pore size distribution curve of Pd-1, Pd-2, Pd-3 and Al_2_O_3_/COR.

The N_2_ adsorption-desorption isotherms and the corresponding BJH pore size distribution curves of Al_2_O_3_/COR, Pd-1, Pd-2, and Pd-3 are displayed in Figure 2. Specific surface area, pore volume, and mean pore size are shown in Table 2.

Figure 2a shows that all samples exhibit type IV isotherms and have an obvious H2 type hysteresis loop in the range of about 0.65–1.0 P/P_0_ (pressure/standard atmospheric pressure), which indicates that the four samples have abundant mesoporous structures on their surfaces. The pore size distributions displayed in Figure 2b show Gaussian curves centered at 9 nm for the four samples. As shown in Table 2, the specific surface area, pore size and pore volume of Al_2_O_3_/COR are higher than Pd-1, Pd-2, and Pd-3. We have ascribed this to two reasons: (1) some Pd species entered the mesoporous channel during loading and resulting in partial mesoporous blockage; and (2) the acidic or alkaline impregnation solution corroded the neutral γ-Al_2_O_3_ coating structure and caused a small part of the pores to collapse. The mass transfer and diffusion of VOCs molecules in the mesoporous channel is relatively rapid. They can successfully contact the active center on the surface of the catalysts to react, and then leave quickly. To sum up, the specific surface area, pore volume and pore size of Pd-1, Pd-2, and Pd-3 catalysts are similar, which means that the mass transfer and diffusion ability of VOCs in these three catalysts are almost the same, and only the active sites can affect the catalytic efficiency.

**Figure 3:**
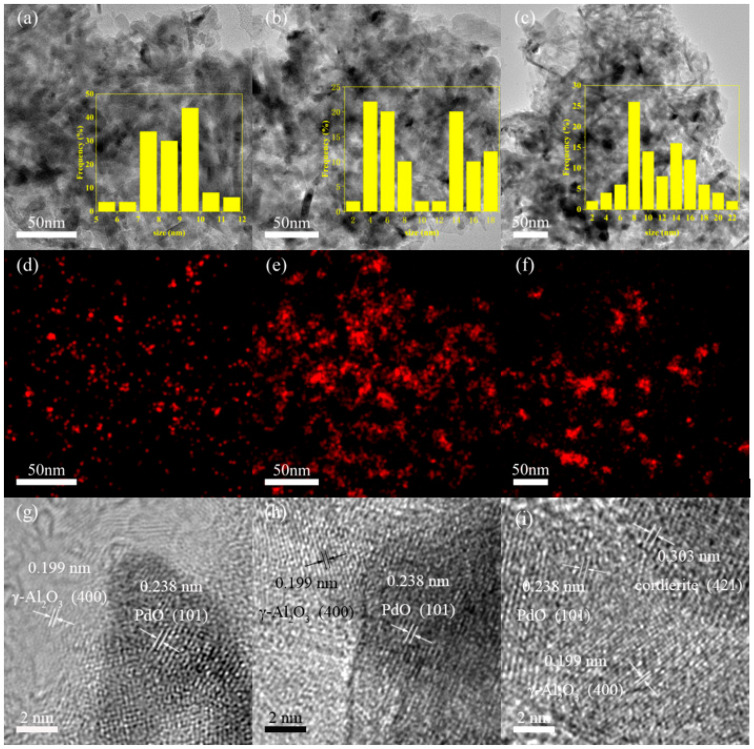
Transmission electron micrographs (**a**–**c**), Pd elemental maps (**d**–**f**) and High-resolution transmission electron micrographs (**g**–**i**) of Pd-1, Pd-2 and Pd-3.

In order to observe the distribution of Pd species, Pd-1, Pd-2 and Pd-3 were characterized by TEM, HRTEM, and EDS. The results are shown in Figure 3. The sizes of PdO nanoparticles (NPs) in Pd-1, Pd-2, and Pd-3 were measured and counted, and the results are presented in the insets of Figure 3a–c. As shown in Figure 3a and its column chart, the main size of PdO NPs in sample Pd-1 was between 7–10 nm, while the size distribution in Pd-2 and Pd-3 is less uniform. For Pd-2, the particle size was mainly below 8 nm and above 14 nm. The particle size distribution of Pd-3 is rather wide, from 2 nm to 22 nm. It can be judged that the dispersion of Pd species in Pd-1 was higher while in Pd-2 and Pd-3 were lower, and the PdO NPs were aggregating in Pd-2 and Pd-3. Meanwhile, the EDS mapping in Figure 3d shows that Pd species in Pd-1 were uniformly distributed on the support, while it was irregularly dispersed in Pd-2 (Figure 3e) and Pd-3 (Figure 3f). This is consistent with the results of XRD.

During the process of calcination, Cl species will combine with Pd species promote the formation of small-size Pd species and improved the dispersion of Pd [20]. However, the calcination temperature used in this work is too high (873 K) that the metal species will not stably adsorb on the surface of the catalyst support. On the other hand, the smaller precious metal NPs have higher surface energy, and they can aggregate through migration and combination of microcrystals, thus forming larger clusters [21]. Therefore, the dispersed Pd NPs in sample Pd-2 will migrate. When they encounter other Pd NPs, they combine with each other to form larger clusters, otherwise they remain small. For Pd-3, the NH_3_ in the precursor Pd (NH_3_)_4_Cl_2_ will gradually leave during the heating process. The frequent change of molecular structure leads to insufficient control of the size of Pd NPs by Cl species, so the particle sizes were different. As shown in Figure 3g–i, the inter-reticular distance of Pd-1, Pd-2, and Pd-3 was measured on the HRTEM, which corresponds to the [400] of γ-Al_2_O_3_ and the [101] of PdO. The cordierite exposed [421] crystal plane was also observed in Pd-3.

The aggregation of Pd species will lead to a significant reduction in the number of active sites for VOC catalytic oxidation. Therefore, the catalytic oxidation activities of the catalysts are limited. Although NO_3_^−^ in Pd (NO_3_)_2_ cannot control particle size, Pd species can spontaneously form particles of more appropriate size, which can provide enough active sites without causing a large number of aggregations due to high surface energy. In addition, the presence of Cl species will poison Pd, thus affecting the catalytic activity of the catalysts [22].

**Figure 4:**
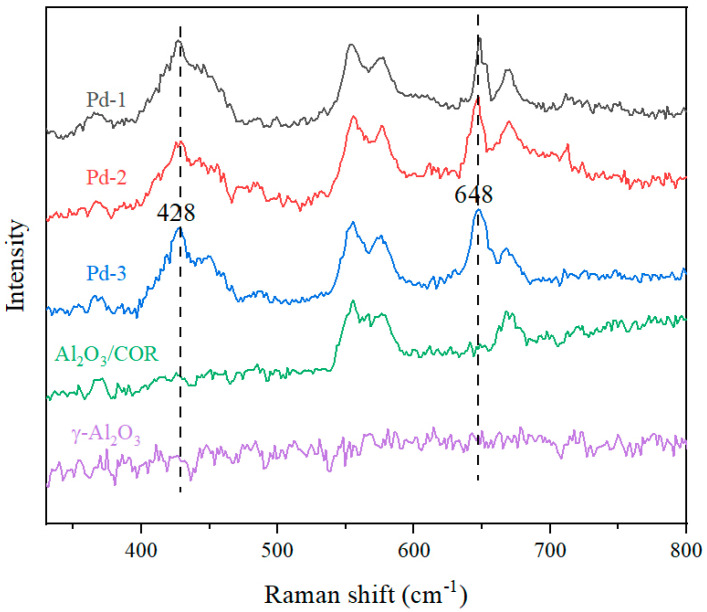
Raman spectra of Pd-1, Pd-2, Pd-3, Al_2_O_3_/COR and Al_2_O_3_.

To gain a better understanding of the catalysts, Pd-1, Pd-2, Pd-3, Al_2_O_3_/COR, and Al_2_O_3_ were characterized by Raman spectroscopy. The results are shown in Figure 4. Pd-1, Pd-2 and Pd-3 samples all contain cordierite and γ-Al_2_O_3_, so the Raman spectra of Al_2_O_3_/COR and Al_2_O_3_ are used for comparison. The sample Al_2_O_3_ has no band between 330 cm^−1^ and 800 cm^−1^, and Al_2_O_3_/COR showed bands at 555 cm^−1^, 575 cm^−1^, and 668 cm^−1^, which all arose from cordierite. Compared with Al_2_O_3_/COR, Pd-1, Pd-2, and Pd-3 loaded with Pd species showed different characteristic bands at 428 cm^−1^ and 648 cm^−1^. The band at 428 cm^−1^ belongs to the E_g_ of Pd-O bond, while the band at 648 cm^−1^ is proved to be B_1g_ of Pd-O bond [23,24]. These two Pd-O bonds do not belong to bulk PdO. It is considered that PdO is obtained by oxidation of Pd species, which attached to the surface of γ-Al_2_O_3_, and can be used for catalytic oxidation of VOCs molecules at once.

### 3.3. Characterization of Metal Dispersion

The adsorption of CO by noble metals is monolayer, and the higher the dispersion of precious metals with the same content, the higher the adsorption capacity of CO. Pulse-CO chemisorption was employed to determine Pd dispersion of Pd-1, Pd-2, and Pd-3. The results were shown in Table 3.

As shown in Table 1, very similar Pd loading was associated with Pd-1, Pd-2 and Pd-3. The higher the metal dispersion, the more active centers the catalysts can provide. As can be seen from Table 3, the metal dispersion of samples Pd-1, Pd-2, and Pd-3 are correspondingly 17.74%, 2.68%, and 4.38%. Evidently, Pd-1 has the highest metal dispersion and the most catalytic active centers. This is consistent with the results of TEM.

### 3.4. Reductive Performance Characterization

**Figure 5:**
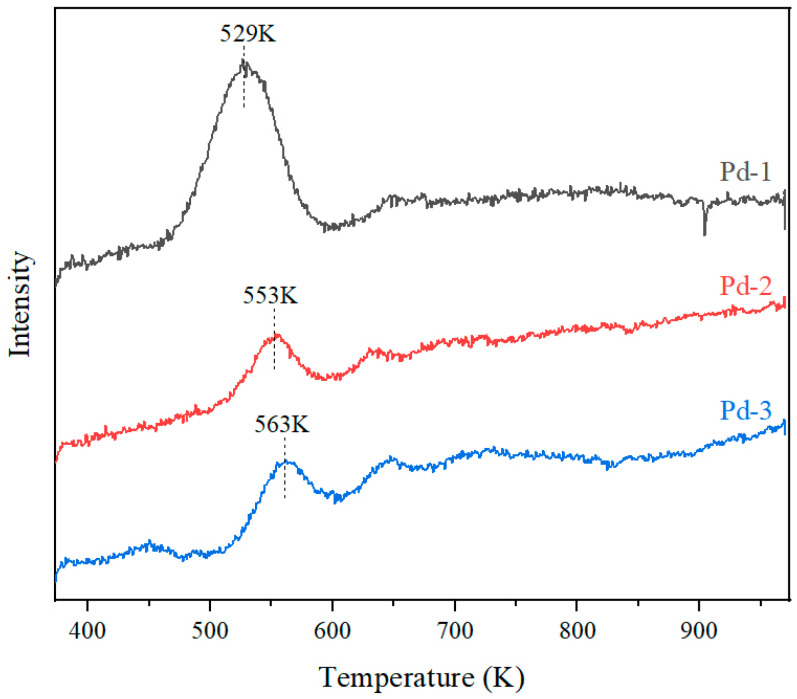
H_2_-TPR diagram of Pd-1, Pd-2 and Pd-3.

The catalytic oxidation performance of catalysts is related to the reduction performance of oxygen species on the active component. The lower the reduction temperature of oxygen species and the greater the reduction amount, the better the active oxygen supply performance and the higher the catalytic oxidation activity of the catalysts [25]. The reducibility of metal oxides in the catalysts can be measured by the temperature programmed reduction of hydrogen. It is generally believed that the lower the reduction temperature of the active components and the greater the hydrogen consumption, the better the active oxygen supply performance of the active components [7]. To estimate the reducibility of Pd-1, Pd-2, and pd-3, H_2_-TPR measurements were conducted, and the results are shown in Figure 5.

Cordierite have no catalytic activity and there is no reactive oxygen species on the surface of Al_2_O_3_, so the peaks of Pd-1, Pd-2 and Pd-3 belong to the reduction behavior of Pd species [26]. As shown in Figure 5 that the reduction temperature of Pd-1 is the lowest, which is 529 K, and the reduction peak is the highest. The peaks of Pd-2 and Pd-3 are lower, and the reduction temperatures are 553 K and 563 K, respectively. It can be concluded that the reduction ability of sample Pd-1 is the best, while that of Pd-2 and Pd-3 are slightly weaker. Combined with the results of ICP-OES, TEM, EDS mapping, and pulse CO chemisorption, it is considered that the Pd species particles in Pd-2 and Pd-3 have smaller surface than that of Pd-1, so fewer effective active sites are exposed by them, resulting in weaker reduction ability of Pd-2 and Pd-3.

### 3.5. Surface Acidity and Valence Analysis

**Figure 6:**
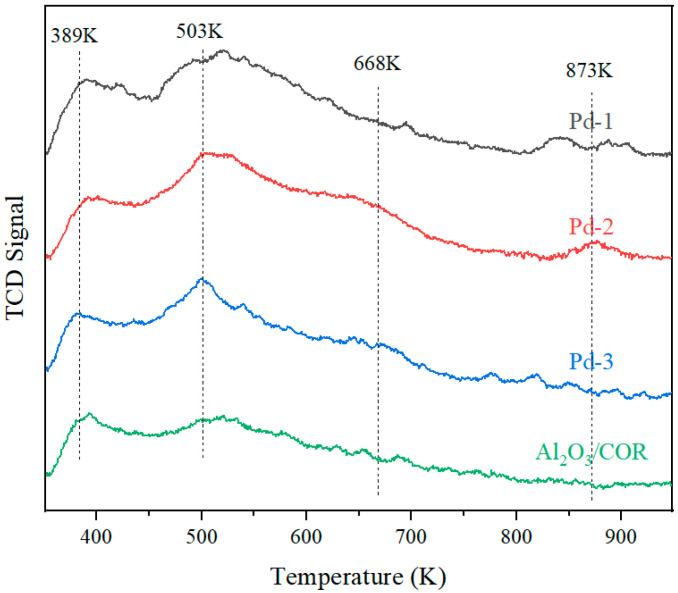
NH_3_-TPD of Pd-1, Pd-2, Pd-3 and Al_2_O_3_/COR.

The peak temperature of the desorption peak characterizes the strength of the acid site, the higher the peak temperature, the greater the acid strength; the area of the desorption peak represents the number of acid sites in the acid center, and the larger the peak area, the more the number of acid sites. To study the relative strength and distribution of acidity on the surface of Pd-1, Pd-2, Pd-3 and Al_2_O_3_/COR, they were characterized by NH_3_-TPD. The results are shown in Figure 6. The data in Table 4 is obtained by integrating the curve in Figure 6. The ranges of weak acid center, medium acid center and strong acid center are 373–523 K, 523–673 K, and 673–923 K, respectively [25,27].

It was reported that the weak acid sites of the catalysts had suitable adsorption strength for VOCs, which was beneficial to the activation and catalytic oxidation of VOCs [7,28]. In contrast, the adsorption intensity of VOCs on medium and strong acid sites was too high, so the adsorbed VOCs were difficult to be activated, which limited the catalytic oxidation efficiency of VOCs [25,27]. It can be seen from Figure 6 that Al_2_O_3_/COR has acidic sites. Two peaks are observed at 389 K and 503 K, respectively, belonging to weak acid sites. For Pd-1, Pd-2 and Pd-3, their ammonia desorption peaks at 503 K are significantly higher than the peak at 389 K. What’s more, two other ammonia desorption peaks are observed at 668 K and 873 K, belonging to medium-strong acid sites and the strong acid sites, respectively. According to Table 4, the loading of Pd species greatly increased the number of weak acid sites. For medium and strong acid sites, the precursors of Pd-1, Pd-2, and Pd-3 have limited effect on them. Generally, the surface acidity of Pd-1 is most suitable for the adsorption and oxidation of VOCs.

**Figure 7:**
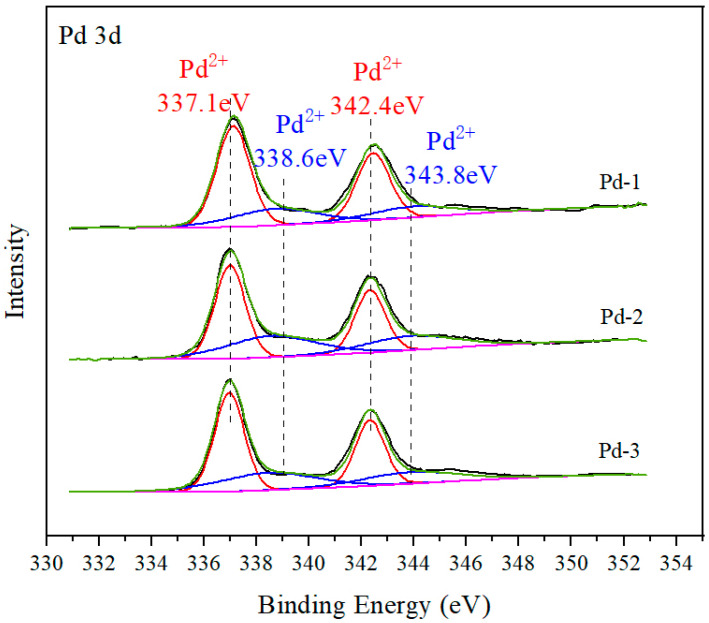
XPS results of Pd 3d in Pd-1, Pd-2 and Pd-3.

To explore the existing form and valence proportion of Pd species in catalysts, Pd-1, Pd-2 and Pd-3 were analyzed by XPS characterization. When using XPS to measure insulators or semiconductors, it is necessary to correct the deviation caused by charge effect. The C1s of external carbon source is usually used as the reference peak for correction. The difference between the measured value and the reference value (284.8 eV) is used as the charge correction value (Δ) to correct the binding energy of other elements in the spectrum. The results are shown in Figure 7. The spectrum of Pd 3d corresponded to spin-orbit splitting into 3d_5/2_ and Pd 3d_3/2_. After deconvolution of XPS patterns, Pd 3d peaks at 337.1 and 342.4 eV were corresponded to Pd^2+^, the peaks at 338.6 and 343.9 eV were corresponded to Pd^4+^ and Pd^0^ was not detected in three samples as no signal at 335.1–335.4 eV is visible in Figure 7. Pd^2+^ species were generally considered to be the active centers in the catalytic oxidation of hydrocarbons [22]. Based on peak area in Figure 7, the proportion of Pd^2+^/(Pd^2+^ + Pd^4+^) was shown in Table 5. Among the three catalysts with the same Pd loading, it was obvious that the Pd^2+^/(Pd^2+^ + Pd^4+^) ratio of the catalysts Pd-1 was the highest. The lower the proportion of Pd^2+^ in Pd-2 and Pd-3, the less effective Pd^2+^ for the catalytic oxidation of VOCs molecules. Pd^2+^ species exist in the catalyst Pd-1, Pd-2 and Pd-3 as PdO.

### 3.6. Mechanism and Products

The Mars-van Krevelen (M_V_K) model was generally used to explain the mechanism of hydrocarbon VOCs oxidation over metal oxides [29,30]. Therefore, it is considered that the MvK mechanism is more consistent with the reaction between Pd-1 and C_2_H_6_. In the MvK model, the oxidation of VOCs is supposed to occur between the lattice oxygen of catalysts and adsorbed VOCs molecules. Adsorbed VOCs react with oxygen species over catalysts, leading to metal oxide reduction. After the reaction, the metal oxide is reduced to metal state. Then, reduced sites are re-oxidized by O_2_ molecules from the feed gas. Therefore, it is PdO that reacts with VOCs and is reduced to Pd^0^. Pd^0^ was oxidized by air again and then participates in the reaction. The final oxidation products of ethane are H_2_O and CO_2_.

We analyzed the tail gas of the fixed bed reactor by gas chromatography, and found that when the reaction temperature was low, the conversion of ethane was low (≤30%), and the oxidation products contained H_2_O, CO, and CO_2_. When the conversion of ethane was high (≥50%), the oxidation products were only CO_2_ and H_2_O.

### 3.7. Catalytic Oxidation Performance

**Figure 8:**
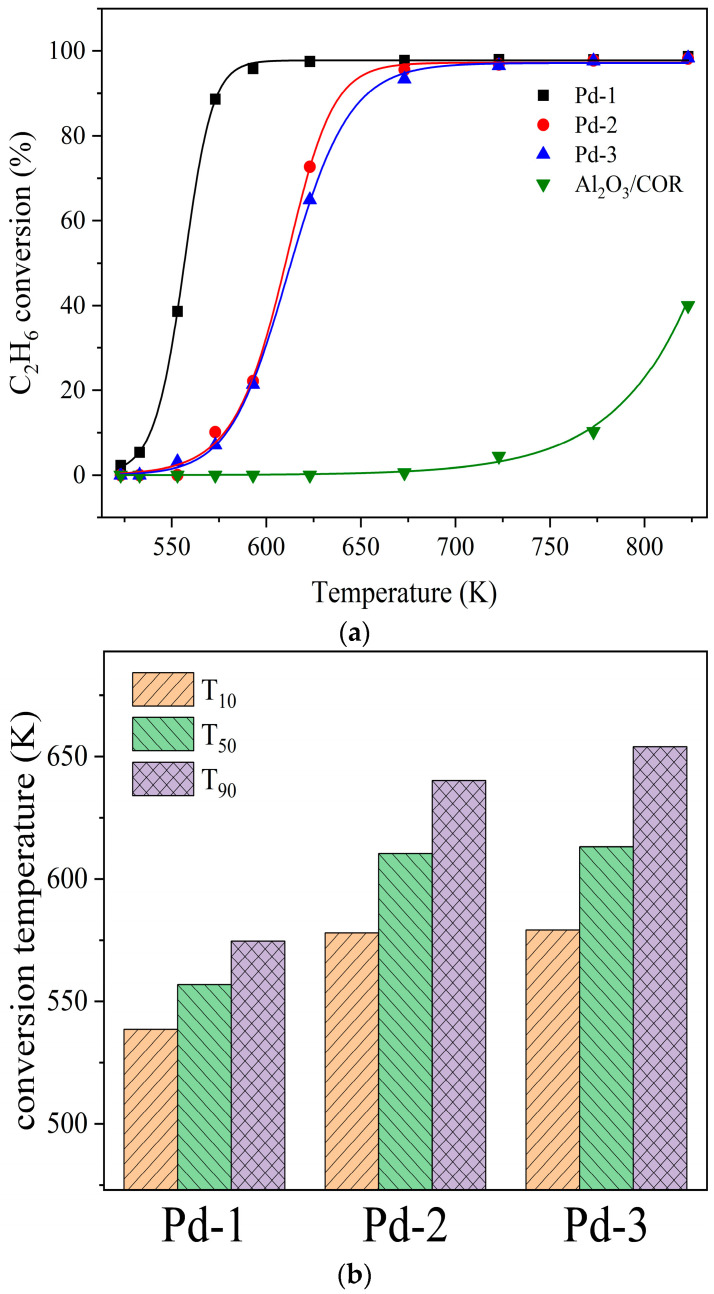
(**a**) Catalytic activity of Pd-1, Pd-2, Pd-3 and γ-Al_2_O_3_/COR for ethane. (**b**) T_10_, T_50_ and T_90_ of Pd-1, Pd-2 and Pd-3.

Pd-1, Pd-2, Pd-3 and Al_2_O_3_/COR were evaluated in a fixed-bed reactor using ethane as a model reaction gas. The results are shown in Figure 8. T_10_, T_50_, and T_90_ are defined as the corresponding temperatures at which 10%, 50%, and 90% of reactant conversions can be obtained, respectively.

The light-off curves of the four samples for C_2_H_6_ oxidation are displayed in Figure 8a, and the detailed activity results are shown in Figure 8b. The sample Al_2_O_3_/COR has almost no catalytic activity. Therefore, the catalytic ability of Pd-1, Pd-2, and Pd-3 comes from their active components. From 523 K to 823 K, Pd-1 has the highest catalytic oxidation activity. As shown in Figure 8b, the T_10_, T_50_ and T_90_ of catalyst Pd-1 are lower than those of Pd-2 and Pd-3. As tabulated in Table 2 and Table 5, Pd-1, Pd-2, and Pd-3 exhibited a similar specific surface area, pore volume and pore diameter and Pd content. Different precursors leading to different particle size and dispersion of active metals have a great influence on the catalytic activity. The higher the dispersion of active metal species, the more active centers for VOC catalytic oxidation, and the higher the activity of the catalysts. The catalyst Pd-1 with Pd(NO_3_)_2_ as the precursor has the best catalytic effect. The comparison of the catalytic activity of Pd-1 and other catalysts for ethane oxidation is shown in Table 6.

**Figure 9:**
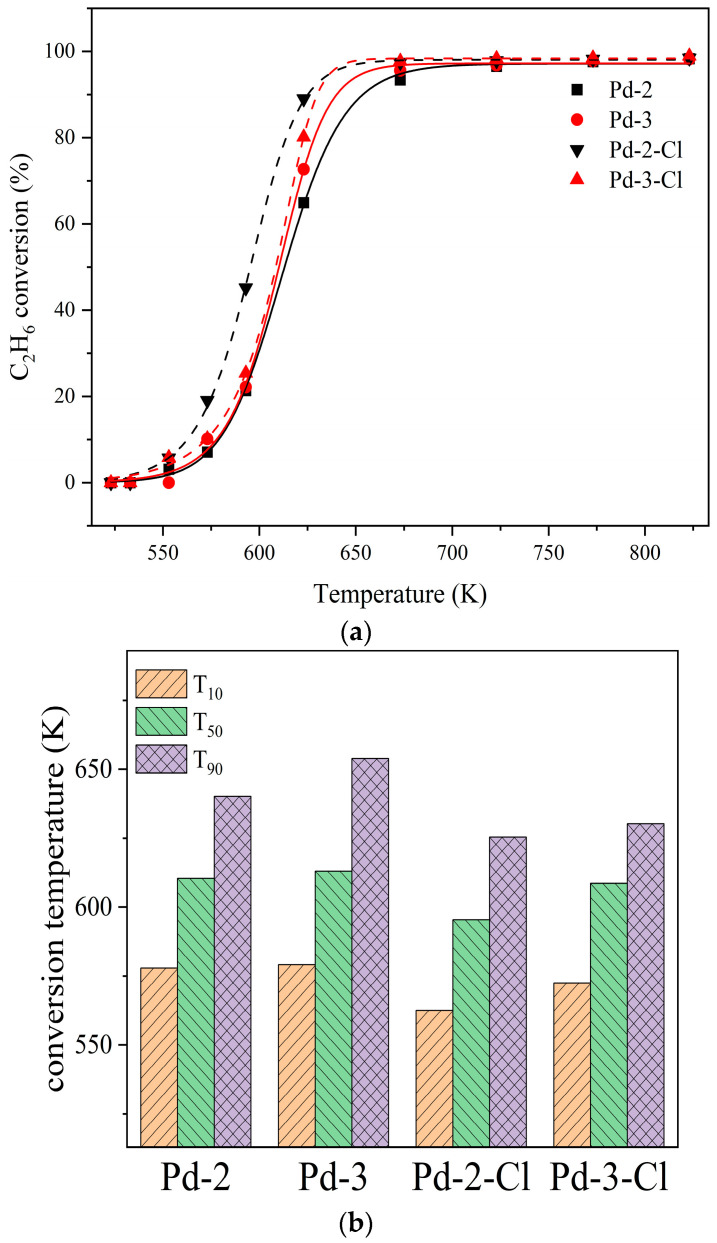
(**a**) Catalytic efficiency of Pd-2, Pd-3 before and after dechlorination. (**b**) T_10_, T_50_ and T_90_ of Pd-2, Pd-3 before and after dechlorination.

As shown in Table 1, the samples of Pd-2 and Pd-3 contain Cl species. In order to verify whether Cl species can inhibit the catalytic activity of Pd-2 and Pd-3, newly prepared catalysts Pd-2 and Pd-3 were dechlorinated by washing and calcined again, which were recorded as Pd-2-Cl and Pd-3-Cl [32]. All experiments including their XRF characterization were carried out using the same methods above and the results obtained are shown in Figure 9 and Table 7.

As can be seen from Table 7, the washing method still has some defects and cannot completely remove the Cl species. By comparing the activity curve before and after dechlorination in Figure 9, the ethane conversion efficiency of Pd-2 and Pd-3 increase by 24.1% and 7.48% after reducing the Cl content in Pd-2 and Pd-3 by 84.8% and 83.9%. The T_10_, T_50_, and T_90_ of Pd-2-Cl and Pd-3-Cl also decreased but their catalytic activities are worse than catalyst Pd-1. The T_10_, T_50_, and T_90_ of Pd-1 are correspondingly 538.6 K, 556.9 K and 574.6 K. These differences are caused by the large particle size of Pd NPs in Pd-2 and Pd-3. Although the root cause was the control effect of Cl species on the particle size, it cannot be remedied by removing Cl species, and can only reduce the effect of catalysts poisoning by Cl species.

**Figure 10:**
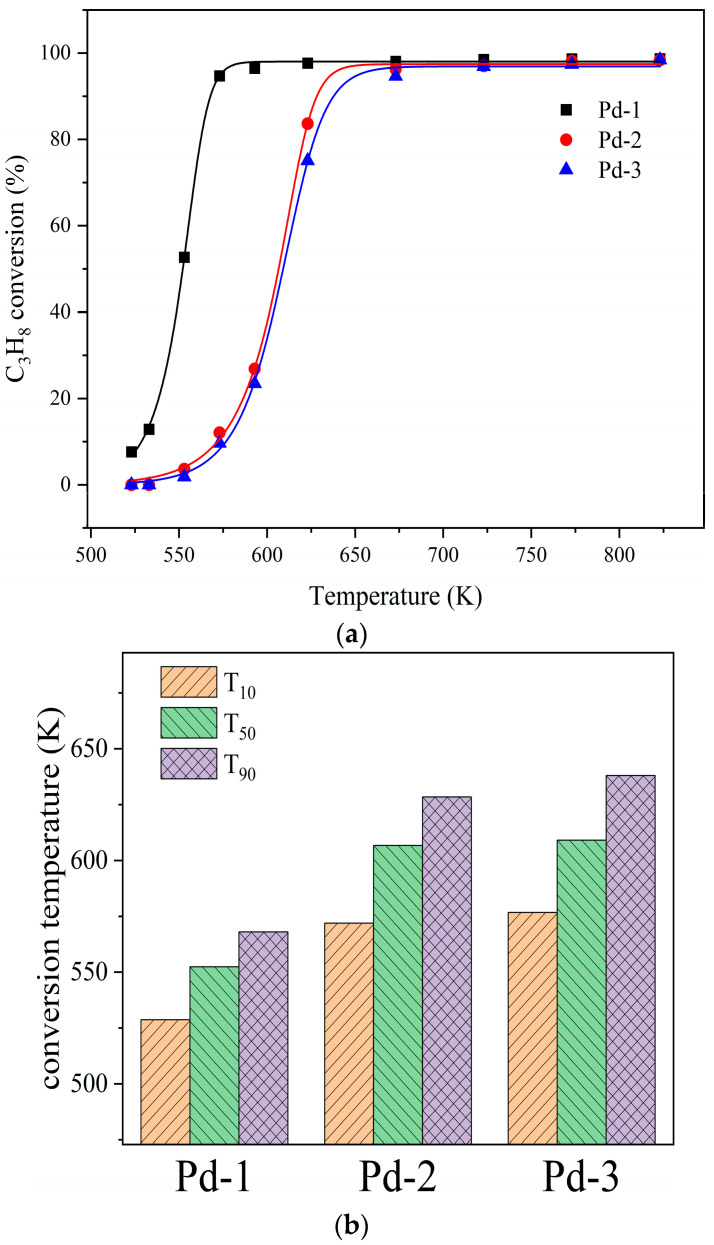
(**a**) Catalytic efficiency of Pd-1, Pd-2 and Pd-3 for propane. (**b**) T_10_, T_50_ and T_90_ Pd-1, Pd-2 and Pd-3 for propane.

To expand the applicability of the catalysts, the model reactants were replaced with propane, and the above evaluation experiments were repeated. The results of the evaluation are summarized in Figure 10. It can be seen from Figure 10a that Pd-1, Pd-2, and Pd-3 catalysts also have high catalytic efficiency for propane. In connection with Figure 10b, the catalytic efficiency of Pd-1 was still the highest, while Pd-2 and Pd-3 are lower. Both Figure 8 and Figure 10 show that the catalytic efficiency of Pd-1 is better than that of Pd-2 and Pd-3.

### 3.8. Water Resistance and Stability

The feed gas was changed into ethane containing 5% water vapor by volume to study the catalytic efficiency of the catalysts in practical application and the remaining conditions remained unchanged. The catalysts were evaluated again.

**Figure 11:**
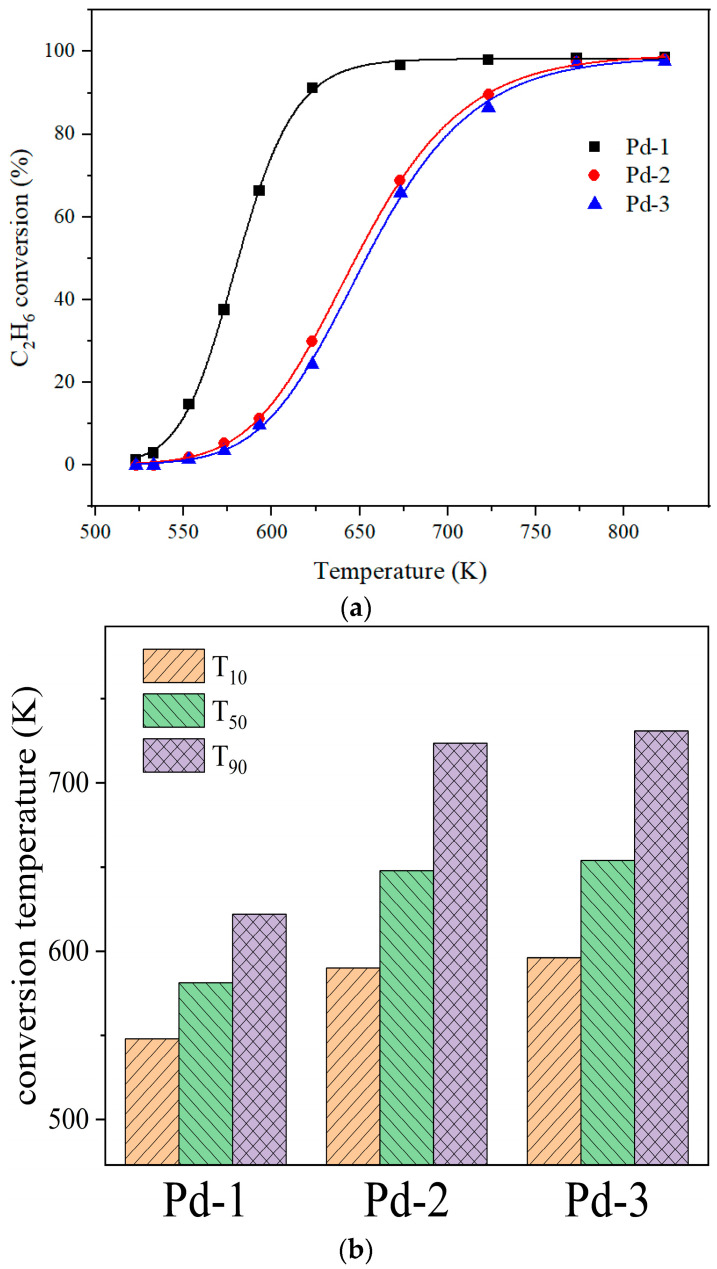
(**a**) Catalytic efficiency of Pd-1, Pd-2 and Pd-3 for ethane in the presence of water vapor. (**b**) T_10_, T_50_ and T_90_ of Pd-1, Pd-2 and Pd-3 for ethane in the presence of water vapor.

Based on the values fo 650 K in Figure 11a, the catalytic efficiency of Pd-1, Pd-2 and Pd-3 for ethane have decreased by 1.39%, 40.7 and 39.9% in aqueous conditions, compared with the results in Figure 8. The water vapor in the feed gas would compete with ethane molecules for the active sites on the surface of the catalysts, and the actual effective active sites for catalytic oxidation decrease. Therefore, under the water-bearing condition, the catalytic effect of the catalysts was weakened, and a higher temperature was needed to help the ethane molecule to catalyze. By comparing Figure 11b to Figure 8b, there is only a small 4.36% increase of T_50_ for Pd-1, and its water resistance was 29.0% and 34.1% better than that of Pd-2 and Pd-3, respectively. Moreover, Pd-1 has the highest catalytic efficiency among the three catalysts, so Pd(NO_3_)_2_ is the most suitable precursor of VOC oxidation catalysts.

**Figure 12:**
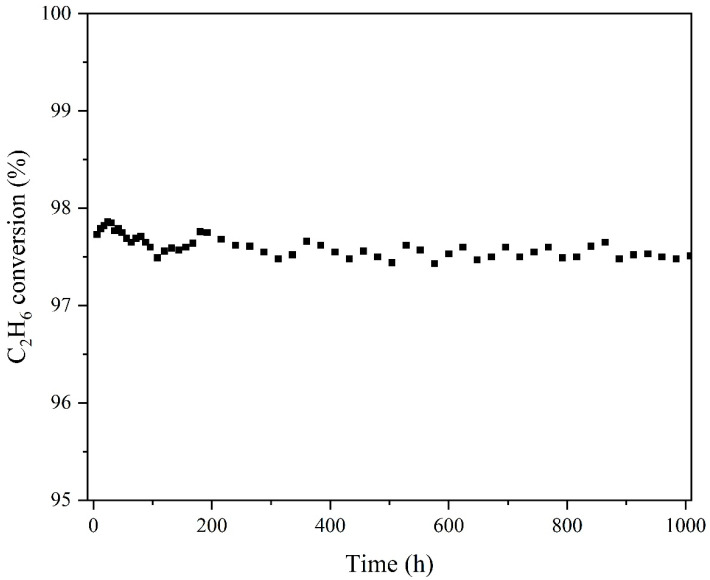
Catalytic efficiency of Pd-1 at 623 K.

To study the long-period stability of the catalyst Pd-1, the long-term evaluation of the catalyst was carried out for 1000 h at 623 K without water vapor. The results are shown in Figure 12. The catalytic efficiency of catalyst Pd-1 for ethane is relatively stable, which always remains between 97% and 98%. The stability may be due to the small particle size and high dispersion of PdO NPs in the catalyst, which inhibits the sintering of the active center in the reaction. In conclusion, Pd-1 not only has high catalytic efficiency and good water resistance, but also shows excellent stability, indicating that Pd-1 with Pd(NO_3_)_2_ as the precursor is an excellent VOCs catalytic oxidation catalysts.

## 4. Conclusions

To sum up, three catalysts were prepared by an impregnation method with Pd(NO_3_)_2_, PdCl_2_, and Pd(NH_3_)_4_Cl_2_ as the corresponding precursors. Based on our experimental setup, a systematic characterization and evaluation showed that the catalyst prepared with Pd(NO_3_)_2_ exhibited a T_90_ at 574.6 K, and a 17.7% dispersion of Pd particles, a uniform size, and a high Pd^2+^ proportion of 70.86%, making it being regarded as the catalyst with the best performance. Compared with Pd-1, the Cl^−^ contained in Pd-2 and Pd-3 can get the particles of Pd species in a small size. In addition, small particles after dispersion are easy to aggregate when the calcination temperature is over 873 K.

The residual Cl^−^ of precursor PdCl_2_ and Pd(NH_3_)_4_Cl_2_ will poison the catalysts and then affect the catalytic oxidation activity of the catalysts. The removal of Cl species can improve the performance of Pd-2 and Pd-3.

It is concluded that Pd(NO_3_)_2_ is the better precursor of VOC oxidation catalyst than PdCl_2_ and Pd(NH_3_)_4_Cl_2_. The issues related to Cl species residues and spontaneous aggregation of small Pd species particles must be addressed before PdCl_2_ and Pd(NH_3_)_4_Cl_2_ can be considered as precursors for developing VOC catalytic oxidation catalysts.

## Figures and Tables

**Table 1:** XRF and ICP-OES results of catalysts Pd-1, Pd-2 and Pd-3.

Catalyst	XRF (Pd)	XRF (Cl)	ICP-OES (Pd^2+^)
Pd-1	0.189% *w*/*w*	-	0.184% *w*/*w*
Pd-2	0.198% *w*/*w*	0.099% *w*/*w*	0.193% *w*/*w*
Pd-3	0.186% *w*/*w*	0.087% *w*/*w*	0.183% *w*/*w*

**Table 2:** Characterization data of nitrogen adsorption and desorption of samples.

Catalyst	S_BET_ (m^2^/g)	V_BJH_ (cm^3^/g)	D_BJH_ (nm)
Al_2_O_3_/COR	16.5	0.0372	9.09
Pd-1	12.2	0.0298	8.63
Pd-2	11.9	0.0293	9.03
Pd-3	12.6	0.0299	8.64
γ-Al_2_O_3_	140	0.482	12.3

**Table 3:** Results of metal dispersion of catalysts Pd-1, Pd-2 and Pd-3.

Catalyst	Metal Dispersion
Pd-1	17.7%
Pd-2	2.68%
Pd-3	4.38%

**Table 4:** Surface acidic sites of Pd-1, Pd-2, Pd-3 and Al_2_O_3_/COR.

Catalyst	Weak Acid Sites (mmol/g)	Moderate Strong Acid Sites (mmol/g)	Strong Acid Sites (mmol/g)
Pd-1	0.0818	0.0061	0.0090
Pd-2	0.0737	0.0177	0.0078
Pd-3	0.0743	0.0093	0.0049
Al_2_O_3_/COR	0.0533	0.0076	0.0035

**Table 5:** Proportion of Pd^2+^ species to total Pd species in catalysts Pd-1, Pd-2 and Pd-3.

Catalyst	Pd^2+^/(Pd^2+^ + Pd^4+^)
Pd-1	70.86%
Pd-2	59.38%
Pd-3	63.68%

**Table 6:** Comparison of catalytic activity of supported noble metal catalysts for ethane oxidation.

Catalysts	Toluene (ppm)	GHSV	T_50_ (K)	T_90_ (K)	Ref.
Au/CoO_X_	5000	15,000 h^−1^	-	523	[31]
Pt/CZ11	1000	50 L g^−1^ h^−1^	543	581	[26]
Pd-1	2000	10,000 h^−1^	556	574	This paper

**Table 7:** XRF results of Pd-2 and Pd-3 before and after dechlorination treatment.

Catalyst	XRF (Cl)
Pd-2	0.099% *w*/*w*
Pd-3	0.087% *w*/*w*
Pd-2-Cl	0.015% *w*/*w*
Pd-3-Cl	0.014% *w*/*w*

## Data Availability

The data presented in this study are available on request from the corresponding author.
